# Selection of private or public hospital care: examining the care-seeking behaviour of patients with private health insurance

**DOI:** 10.1186/s12913-020-05253-y

**Published:** 2020-05-06

**Authors:** Rezwanul Hasan Rana, Khorshed Alam, Jeff Gow

**Affiliations:** 1 School of Commerce, University of Southern Queensland, Toowoomba, Australia; 2 School of Commerce, Centre for Health Research, University of Southern Queensland, Toowoomba, Australia; 3 School of Accounting, Economics and Finance, University of KwaZulu-Natal, Durban, South Africa

**Keywords:** Australia, Care-seeking, Healthcare use, Hospitals, HILDA, Private health insurance

## Abstract

**Background:**

This study aimed to examine the healthcare-seeking (hospital, primary and preventive care) and healthcare utilisation behaviour of patients with private health insurance (PHI) in Australia. It also aimed to examine the socioeconomic, demographic and lifestyle factors that influence the choice of hospital care in Australia.

**Method:**

A logistic regression model with repeated measure t-test and Pearson’s Chi-square test were used to identify the factors that affect the choice of care. Data from waves 9 (2009) and 13 (2013) of the nationally-representative Household, Income and Labour Dynamics in Australia (HILDA) survey were used in the analysis.

**Results:**

Patients with PHI had a higher number of hospital nights’ stay despite having a lower number of hospital admissions than those without private cover. Significant disparities were identified in preventive and specialist care use between patients with cover and without cover. No significant variations were observed in healthcare utilisation for PHI patients before and after dropping PHI. One in four patients chose to use public hospitals despite holding PHI cover. Moreover, those insured and from lower socioeconomic backgrounds and those who were younger and without long-term health conditions showed a higher probability of selecting public rather than private care.

**Conclusions:**

It is evident that PHI cover encourages people to use private care. However, a considerable number of PHI patients are using public care, even though eligible for private care may indicate consumer information asymmetry.

## Background

In the emergency department of Australian public hospitals, patients with private health insurance (PHI) are asked to decide whether they want to be treated as public or private patients. Interestingly, for people with PHI cover, the answer is not always obvious. The policies promoting PHI in Australia often focus on increasing its attractiveness to promote private health care usage and thus reduce pressure on the public system [1]. A recent report published by the ‘Senate Community Affairs Reference Committee’ found that patients are often unaware of the potential out-of-pocket treatment costs when using the private health system [2]. Many patients with PHI cover do not opt for private hospital care but instead end up in public hospitals undermining the policy aim of redirecting public hospital demand to the private sector. Higher enrolment rates for PHI will not save scarce public resources unless the PHI system encourages those patients to use private hospitals solely. In addition, a PHI system that promotes unequal access to care is also undesirable. Hence, to improve overall outcomes in the health care system, it is imperative to understand the factors influencing the choice facing patients with PHI and their use of medical care services in Australia.

Previous studies related to PHI in Australia have mainly focused on the factors determining patients’ decision to purchase PHI cover [3, 4], the adverse selection problem (at a given premium high-risk individuals will have more incentive to purchase PHI than low-risk individuals) [5] and whether PHI increases utilisation of hospital care [6] and other medical treatments [7]. Others argued for [3, 6] and against [1, 8, 9] the justification of providing public subsidies to take up PHI via tax rebates and other fiscal incentives. Little is known regarding the hospital and preventive care-seeking attitudes of patients with and without PHI cover in Australia. Moreover, it is still unclear what socioeconomic and demographic factors influence patients with PHI cover to access public hospitals as a public patient despite paying for and having the availability of private hospital care. Lastly, to the best of authors’ knowledge, no study has yet examined the differences in healthcare utilisation for patients who held and then dropped PHI cover. A nationally representative survey data set is used to examine these issues.

To address these gaps in the literature, this paper aims to examine the disparity in healthcare use of individuals with and without PHI cover and to identify the socioeconomic, demographic, geographic and lifestyle characteristics that influence the choice of hospital care (public vs private) of patients with PHI. Equality of access is a major goal of the Australian health system through Medicare, the national health insurance scheme. Yet simultaneously, public resources are directed towards individuals and organisations to promote private healthcare which is in conflict with that aim. There seems to be little justification for promoting PHI if it does not considerably reduce public sector demand. The findings of this study will assist in the discussion of the optimal policy mix to address the issues of access and equity in the Australian hospital system.

This study will add to the existing literature by answering the following research questions: i) to what extent does the hospital care-seeking attitudes and use of secondary preventive and specialist care vary between those with or without PHI cover?; ii) what factors influence the choice of the type of hospital care (public vs private) among patients with PHI cover? and iii) does healthcare use differ significantly for individuals before and after dropping PHI cover?

These issues are particularly important concerns for countries where universal public healthcare is supplemented by a privately funded health system (e.g. Australia, Ireland, Canada and the UK). The findings will assist policymakers to realise whether current healthcare policy settings which promote PHI are effective in reducing demand for public hospital care. Further analysis will reveal whether PHI cover encourages people to consume additional healthcare services. Moreover, understanding the factors influencing hospital care-seeking behaviour of patients with PHI cover will offer policy guidance based on consumer demand and actual use of health services.

### Australian healthcare system in brief

The study setting of this paper is Australia, a developed country that has a sound and relatively sophisticated healthcare system which ranks very high internationally and also amongst the Organisation for Economic Co-operation and Development (OECD) countries [10, 11]. The population of Australia enjoys higher life expectancy, lower infant mortality and fewer disability-adjusted life years compared to the OECD country average while the share of national healthcare expenditure to gross domestic product (GDP) is at the median among OECD countries [12]. Residents of Australia (in 2018) had a higher average number of doctor consultations and a lower average length of stay in hospital and fewer waiting days for elective surgery compared to the average in OECD countries [13]. For instance, the median waiting days for Cataract surgery, Coronary bypass and Hip replacement in Australia were 85 days, 13 days and 110 days, respectively, compared to the OECD average of 103 days, 22 days and 128 days. Hence, if health status and use of healthcare services are principal indicators of the performance of a healthcare system, Australia’s health sector is doing an efficient job in comparison to other OECD countries.

The federal, state and territory governments of Australia share the responsibility to finance, develop and implement policies, and regulate and monitor the healthcare system. The health system is a multi-layered network of public and private service providers and supporting mechanisms [12]. Healthcare is provided through general practitioners (GPs) (primary care services), medical specialists, allied health workers, hospitals, nurses and other health professionals.

The universal tax-funded public health insurance program in Australia is called ‘Medicare’. It has three major parts: medical services, public hospitals and medicines. It covers the expenses of public hospital services (free treatment for patients in public hospitals) and visits to doctors (payment of benefits or rebates for using selected professional healthcare services through the ‘Medicare Benefits Schedule’) [14]. Further, the ‘Pharmaceutical Benefits Scheme’ provides subsidies for a variety of prescription medicines. Hence, the fundamental structure of the hospital and medical services has been established in a way to provide essential healthcare services to all Australians without experiencing financial hardship [15].

The Australian healthcare system is often called a hybrid model because, in addition to Medicare, people can also purchase private health insurance to gain access to both public and private hospitals as private patients [16] and extra coverage of services (e.g. dental care and physiotherapy), items not covered by Medicare [17]. Australian health policy encourages private health cover (through tax incentives or monetary rebates on premiums) so that private hospital care can complement (but sometimes duplicate) the services provided by public hospitals. The aim is to reduce public healthcare expenditure and improve access to and quality of the public health sector [18]. Hence, promoting PHI is a pivotal mechanism to manage the rising burden of healthcare demand for the rapidly ageing Australian population. Moreover, PHI also provides patients with more options regarding their choice of doctors and type of services [6]. Nonetheless, the policy of subsidising private health insurance through the tax system is a contentious issue, and some argue that it creates inequality in access to care [19].

**Fig. 1:**
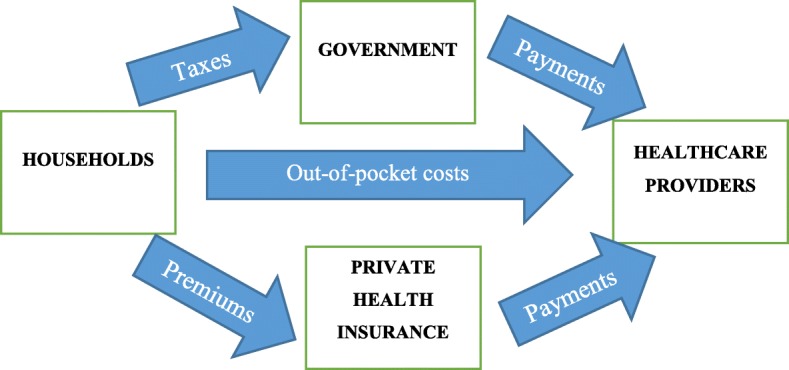
Flow of health funding in Australian health sector**.** Source: Duckett and Willcox (2015)

Healthcare in Australia is financed using a mixture of public and private sources [17]. According to the Department of Health in 2016–17, 41% of healthcare costs were financed by the Federal Government, 27% by the six state and two territory governments, 17% by individuals, 9% by private health insurers and 6% by non-government organisations [20]. Eventually, all healthcare spending is financed by households through taxation, out-of-pocket expenditure or private health insurance premiums [21]. In total, there were 695 public and 630 private hospitals in Australia (in 2016) [22]. Figure 1 provides a basic health funding flowchart of the Australian health sector. It is important to note that some households contribute more than others, and some utilise healthcare services more than they contribute. Hence, the health financing mechanism is redistributive and focused on achieving equity in access, regardless of socioeconomic status.

**Fig. 2:**
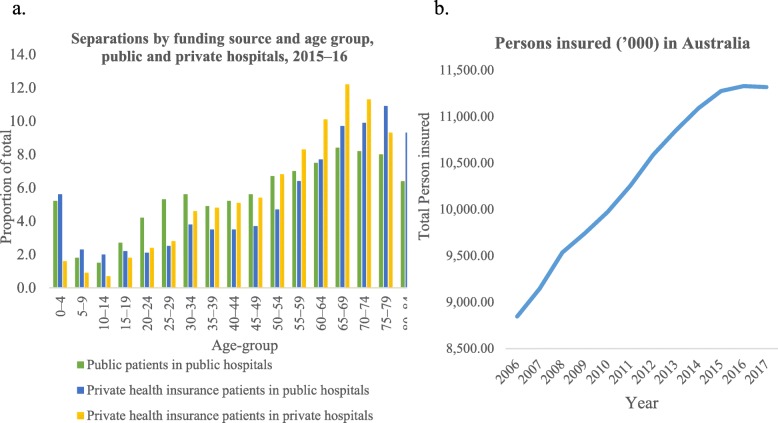
Trends in the number of persons insured and choice of hospital care in Australia. Source: AIHW (2018). Australian Institute of Health and Welfare. Data available from: https://www.aihw.gov.au/reports/hospitals/private-health-insurance-use-hospitals/data

Figure 2 shows the choice of hospital care type by age group and the trends in the number of persons insured in Australia. Expectedly, as age increases, so does the use of all types of hospital care. The propensity for the selection of private care in preference to public care, increases at an increasing rate after the age of 50 (Fig. 2). On the other hand, Fig. 2 also indicates the decreasing trend of the total number of people with PHI cover. Further information on the current state of PHI and Australian hospitals is available from a recent report published by the Australian Institute of Health and Welfare (AIHW) [23].

### The conceptual framework

This study seeks to understand the relationship between PHI status and the medical care-seeking attitude of patients with PHI cover in Australia. Having PHI cover is desirable as it provides patients with more choices regarding doctors, type of services and reduced waiting times while protecting patients from additional healthcare expenditures not covered by ‘Medicare’ [3, 6]. Hence, following van Gameren [24], the consumption of health services by a patient (with PHI cover) from a utility maximisation perspective can be divided into two parts: consumption of publicly (H_pb_) and privately funded healthcare (H_pt_). If C is the consumption of all other goods and M is the total income then, the utility maximisation function restricted by income (total expenses are not higher than income) is,

$$ \operatorname{Max}\ U\ \left(\mathrm{C},{\mathrm{H}}_{\mathrm{pb}},{\mathrm{H}}_{\mathrm{pt}}\right) $$

$$ \mathrm{M}\ge {\mathrm{P}}_{\mathrm{pb}}{\mathrm{H}}_{\mathrm{pb}}+{\mathrm{P}}_{\mathrm{pt}}{\mathrm{H}}_{\mathrm{pt}}+{\mathrm{P}}_{\mathrm{c}}\mathrm{C} $$

where P_pb_ is the price of public health services, P_pt_ is the price of private health services, and P_c_ is the price of all other consumption goods. Although the demand for health services is unique in nature (which depends on individuals’ stock of health and their health problems), it is assumed to be a normal good, which means that holding other things constant, increasing price decreases the demand for health services [25].

Eldridge et al. [6] showed that in a hypothetical scenario if everyone has PHI cover, it reduces the effective price of private healthcare; therefore, the demand for private hospitals will increase, and demand for public hospitals will reduce. This switching of demand from the public to private is logical for a country which does not offer public health insurance for all. However, in Australia, given the existence of Medicare, private health services could be seen as duplicate, complementary and supplementary to public health services [19]. Therefore, the choice of the type of services consumed by patients with PHI cover varies considerably, and increasing the enrolment rate in PHI may not divert demand from the public sector to the private sector at the desired (optimal) level. If the type and quality of services are the same between private and public hospitals, the price elasticity of demand for private hospital services will be high. As services in public hospitals can be consumed at low or no cost, patients will avoid private hospital care even if there is an expectation (not actual) of higher premiums in the future (for utilising private care regularly). The availability of publicly funded health coverage increases the opportunity cost (the relative price P_pt_/P_pb_) of using privately funded services; hence, a patient will be more inclined to consume public hospital care [24].

The model focuses on the impact of PHI on the utilisation of secondary preventive and primary care, and the type of hospital care choices made while taking into account several compounding variables (e.g. age, income and BMI) which might influence the demand for healthcare services.

The rest of this study is structured as follows. The next section explains the data and method. Section three consists of the results of the study, followed by a detailed discussion of the findings. The final section provides a brief conclusion to the study.

## Methods

### Data source and study population

Data were drawn from the ‘Household Income and Labour Dynamics in Australia’ (HILDA) survey wave 9 (2009) and wave 13 (2013). HILDA is a nationally representative longitudinal survey collected annually since 2001, by the Melbourne Institute of Applied Economic and Social Research [26]. The survey is conducted in accordance with the ethical guidelines approved by the University of Melbourne [27]. Therefore, additional ethical approvals were not required for the current study. Data are available for approved users from the Department of Social Services.

Both the selected waves had special additional questions related to the health and personality of respondents. Health-specific questions are only conducted every four years. Only waves 5, 9 and 13 were available (with health-specific questions which were used in this study) when the study was being planned, developed, written and data analysis was conducted.

The total number of persons, in the 7234 responding households in 2009 were 17,632 and from 7463 responding households in 2013 were 23,299 individuals [28]. Data were collected via face-to-face interviews and through a self-completed questionnaire from each household. The detailed methodology of the HILDA survey is outlined in [29]. Along with the general survey data, the health-focused waves of 2009 and 2013 accumulated data on healthcare utilisation (GP and hospital visits), general health and well-being (self-assessed health), lifestyle (physical activity, smoking), the prevalence of chronic disease and PHI status. A person with PHI cover was identified with the following question, ‘apart from Medicare, are you currently covered by private health insurance?’ A total of 13,244 (after excluding missing values) individuals (yes = 7001, no = 6243) had valid responses in 2009 and for 2013 the total number of valid responses were 17,425 (after excluding missing values) (yes = 9676, no = 7749).

### Variable selections and measures

A brief description of variable definition, types and measurements is presented in Table 1. Two key independent variables were identified: the PHI status of an individual and their choice of hospital admission type. For the logistic regression, the dependent variable is measured as follows (for a respondent with PHI cover): hospital admission type = 1 if a public patient in a public hospital and 0 otherwise. In the survey, respondents with current PHI cover were also questioned regarding the type of PHI cover purchased. There are three types of cover; hospital only (covers for the cost of treatments as private patients at the hospitals), ancillary/extras only (covers the cost of services outside of hospitals such as a psychologist) or both. Also, individuals with PHI and who had an overnight hospital stay in the previous 12 months were asked about the ‘hospital overnight admission type’. Individuals had to choose from three options; i) public (Medicare) patient in a public hospital, ii) private patient in a private hospital, iii) private patient in a public hospital. For simplification of the analysis a binary variable (hospital admission type) was created where a person with PHI and selected to be a public patient (treated as a patient without PHI) in a public hospital was coded as 1 and 0 otherwise (private patient in a public hospital or private patient in a private hospital).

**Table 1:** Variable definition

Variable	Variable type	Measurement
**Independent variables (Logistic regression)**		
For respondents with PHI: Hospital admission type	Binary	1 if a public patient in a public hospital and 0 otherwise
For respondents with PHI: Who had an overnight hospital stay	Binary	Selected to be a public patient (treated as a patient without PHI) in a public hospital was coded as 1 and 0 otherwise (private patient in a public hospital or private patient in a public hospital).
**Other explanatory variables**		
Number of doctor visits Number of hospital admissions Number of nights per hospital admission	Continuous	Positive values from 0 to upwards.
Whether during the last 12 months, respondents had: Visited a hospital doctor Visited a specialist doctor Visited a mental health professional Health check-ups or screening	Binary	Yes = 1 No = 0
Household annual expenditure on pharmaceuticals Fees paid to health practitioners	Continuous	Positive values from 0 to upwards.
Household disposable income (DY)	Ordinal	Four categories: Low income is DY<$63,746, lower middle income is DY = $63,746 to $100,757, higher middle income is $100,758 to $144,848 and high income is DY>$144,849. Calculated based on the income level of the respondents of the respective waves.
Age	Ordinal	Three categories: age < 45; age 45–65; age > 65.
Education level	Binary	Two categories: > High school; ≤ High school.
Body Mass Index (BMI)	Ordinal	Four categories based on the respondents BMI: BMI = < 18.5; BMI 18.6–24.9; BMI 25–29.9 BMI= > 30.
Self-assessed health	Scale	Five categories (excellent, very good, good, fair and poor) using scale 1–5.
Prevalence of long-term health conditions	Binary	Yes = 1 No = 0
Marital status	Binary	Two categories: Currently married = 1 and all other situation = 0.
Mental health status	Scale	Kessler psychological distress scale (low, moderate, high and very high) using values 1–4.
Physical activity	Ordinal	Three categories: less than once a week, 1–3 times a week, more than three times per week.
Smoking status (Smokes cigarettes or other tobacco products)	Ordinal	Three categories: non-smoker = I have never or no longer smoke; regular smoker = Yes, I smoke; occasional smoker = all other answers.
Health shocks (Serious personal illness in the last 12 months)	Binary	Yes = 1 No = 0
Financial distress (Major worsening of finances)	Binary	Yes = 1 No = 0
Financial risk-taking attitude	Ordinal	Three categories: never takes risk, takes average risks and takes sizeable risks.
Remoteness	Binary	Two categories: Urban and rural. Using ‘ASGC 2001 Section of State’ variable in the HILDA data as suggested by the Australian Bureau of Statistics.
Full time students	Binary	Yes = 1 No = 0

Several additional variables were used to examine variations in healthcare utilisation between respondents with and without PHI cover. These include the number of doctor visits, number of hospital admissions, and the number of nights stay per hospital admission. Other variables included were whether during the last 12 months respondents had visited a hospital doctor, a specialist doctor or a mental health professional and whether they had health check-ups for breast, prostate or bowel cancer screening, cholesterol or blood pressure during this period. These were also designated as binary variables (yes = 1, no = 0). The level of preventive care utilisation was measured using screening for pap smear, breast cancer, prostate cancer, bowel cancer and cholesterol provided by insured adults in the previous 12 months [30].

Household annual expenditure on pharmaceuticals and fees paid to health practitioners were used to measure out-of-pocket health expenditure. To understand the current state of an individual’s health, three variables were included. Self-assessed health used a Likert scale in five categories (excellent, very good, good, fair and poor) and prevalence of long-term health conditions (yes = 1, no = 0) and mental health status was measured with the Kessler psychological distress scale (low, moderate, high and very high) [31]. Lifestyle variables consisted of physical activity (less than once a week, 1–3 times a week, more than three times per week) and smoking status (non-smoker, occasional smoker, regular smoker). Health shocks (illness) or financial distress can influence the choice of healthcare utilisation [32, 33]. Therefore, health shocks and financial distress were measured thus: serious personal illness (yes =1, no = 0) and major worsening in finances (yes =1, no = 0), either of these in the last twelve months.

Other key variables that have a confounding influence on health and healthcare utilisation such as age, gender, education, income, body mass index (BMI), marital status, remoteness from hospital and birthplace were also utilised [6, 17, 34]. The age range of the respondent population was 15 to 101, and they were divided into three groups (age < 45; age 45–65; age > 65), education level was divided into two categories (> High school; ≤ High school). Furthermore, two subgroups were created for marital status (currently married and all other situations), four groups for BMI and remoteness was calculated using the variable ‘section of the state’ based on the guidelines of the Australian Bureau of Statistics [35]. Moreover, Booth-Kewley and Vickers [36] concluded that personality is a key determinant of health behaviour. To add to the previous literature, this study examined whether financial risk-taking behaviour (a measure of personality) impacts the healthcare-seeking attitude of individuals with PHI cover (never takes financial risk, takes average financial risks and takes sizeable financial risks). Lastly, a dummy variable for full-time students was used to estimate whether not being part of the labour force had an effect on the selection of healthcare services.

### Statistical analysis

Four types of statistical analyses were performed. First, unadjusted descriptive analyses were conducted to estimate the heterogeneity in the type of hospital care and preventive care utilisation based on PHI status. Respondents with PHI cover were further categorised into the three types. Second, repeated measure t-tests were performed for selected sub-groups of participants to examine whether a change in PHI status significantly impacts healthcare utilisation. Using the ‘xwaveid’ indication in the data, a cohort of people were selected who were common to both waves. Next, a sub-group of 193 respondents were identified who had PHI cover in 2009 but had dropped it in 2013. Then, the repeated measure t-tests were used to compare the healthcare utilisation of this sub-group. Third, Pearson’s Chi-square test was used to compare whether the choice of hospital service varied depending on the socioeconomic, demographic, health status and lifestyle characteristics of patients in both waves. Finally, logistic regression was employed to determine the factors influencing hospital choice by different types of patients. This approach is commonly used [37, 38] to predict a categorical (mainly dichotomous) variable with a mix of continuous and categorical predictor variables [37, 39]. The regression model here predicts the probability of admission as a public patient whilst holding PHI cover. The estimation was performed with patients with PHI cover and who had overnight hospital admissions in 2013. The following binary logistic regression model was used:

$$ \log \frac{Y_p}{\left(1-{Y}_p\right)}={a}_0+{\sum}_{n=1}^q\left({\beta}_n{x}_{pn}\right)+{u}_p $$

where Y is the binary dependent variable and *Y*_*p*_ is the probability of a patient with PHI cover choosing the option of being a public patient in a public hospital. *x*_*pn*_ are the predictor variables for *p*^*th*^ observations, *β*_*n*_ are the estimated coefficients and *u*_*p*_ indicates error-terms.

Tests statistics were calculated using bootstrap methods based on 1000 draws, which reduce biases from lack of normality and homoscedasticity [39, 40].

For a robustness check, regression analysis was conducted adding state dummies (Australian Capital Territory as the reference category) in the model to control for potential state-wise variations in PHI policies, systems and practices.

## Results

### PHI status and healthcare utilisation

Table 2 shows the percentage of respondents who used different types of hospital care and had health check-ups (secondary preventive care) between 2009 and 2013. Overall, around 75% of patients with PHI cover selected the private patient option, and the rest consumed public hospital services as a public patient. Hence, almost a quarter of the respondents preferred publicly funded services despite having PHI cover. Conversely, around 7 to 9% of patients without PHI cover preferred to be a private hospital patient. As expected, there are significant differences in the type of hospital care consumed for patients with only ancillary/extras cover and those with a hospital cover. Patients with PHI preferring public patient care and no cover patients preferring private care reduced by 3 and 2%, respectively, but the rate of health check-ups remained the same between the two waves.

**Table 2:** Public vs private care utilisation by health insurance status, type of cover and membership (%)

	*Public patient in public hospital*	*Private patient in private hospital*	*Private patient in public hospital*	*Health check-up in last 12 months*	*Public patient in* *public hospital*	*Private patient in private hospital*	*Private patient in public hospital*	*Health check-up in last 12 months*
*YEAR*	***2009***				***2013***			
**PHI status**								
Yes	25.3	58.0	16.4	75.2	22.7	59.9	17.8	75.7
No	90.7	6.7	2.3	67.6	92.8	4.9	2.1	68.6
**PHI cover type**								
Hospital only	26.3	56.1	17.5	77.0	21.7	59.0	19.4	78.5
Extras only	91.9	6.5	1.6	74.6	90.5	3.2	6.3	68.8
Both	18.9	63.3	17.6	75.4	18.3	63.1	18.5	76.3
**Membership type**								
Family	25.2	60.0	14.6	69.8	24.3	59.7	15.9	70.0
Couple	19.8	62.1	18.1	87.2	18.8	60.4	20.8	87.1
Single	29.1	53.0	17.8	78.3	22.3	59.1	18.4	78.3

Note: Values in percentage. 2009 and 2013 are data from Wave 9 and Wave 13, respectively. Services used in the last 12 months prior to the date interviewed. Public patient in public hospital means a person with no PHI using public hospital services; private patient in private hospital means a person with PHI using private hospital services; private patient in public hospital means a person with PHI using public hospital services

Table 3 presents data on healthcare use and health screening by respondents with PHI cover (excluding ancillary cover only) and no cover from 2009 and 2013. On average, patients with PHI cover had slightly longer overnight stays (1.82 vs 1.74 in 2009 and 1.85 vs 1.76 in 2013, *p* < 0.05) despite having a significantly lower number of hospital admissions and doctor visits than those with no cover. Having PHI cover is also significantly related to a higher number of specialist doctor visits (0.51 vs 0.48 in 2009 and 0.52 vs 0.47 in 2013, *p* < 0.05). Noticeably, respondents with PHI cover reported a higher level of health screening (e.g. Breast screening: 0.21 vs 0.14 in 2009 and 0.20 vs 0.14 in 2013, p < 0.05) compared to no cover respondents, and the mean differences are significant at a 95% confidence interval.

**Table 3:** Differences in healthcare use between individuals with PHI and no PHI

Variables	2009		2013	
Healthcare use (last 12 months)	Cover	No cover	Cover	No cover
Number of doctor visits	5.63^a^ (.102)	8.51 (.187)	5.55^a^ (.081)	8.54 (.163)
Number of hospital admissions	0.26^a^ (.010)	0.45 (.028)	0.28^a^ (.011)	0.50 (.049)
Patient in a hospital overnight	1.82^a^ (.006)	1.74 (.008)	1.85^a^ (.005)	1.76 (.007)
Specialist doctor visits	0.51^a^ (.008)	0.48 (.009)	0.52^a^ (.006)	0.47 (.008)
Mental health professional	0.08^a^ (.004)	0.11(.006)	0.10^a^ (.004)	0.15 (.006)
*Health screening*				
Pap smear	0.29^a^ (.006)	0.25 (.008)	0.28^a^ (.006)	0.24 (.007)
Breast screening	0.21^a^ (.007)	0.14 (.006)	0.20^a^ (.005)	0.14 (.006)
Prostate check	0.16^a^ (.006)	0.12 (.006)	0.14^a^ (.005)	0.12 (.005)
Screening for bowel cancer	0.16^a^ (.006)	0.12 (.006)	0.17^a^ (.005)	0.13 (.006)
X-rays	0.25^a^ (.007)	0.32 (.007)	0.26^a^ (.006)	0.32 (.008)
Blood pressure	0.75 (.007)	0.75 (.007)	0.77 (.005)	0.76 (.007)
Cholesterol test	0.52^a^ (.008)	0.45 (.009)	0.52^a^ (.006)	0.48 (.008)

Notes: Respondents with cover = 5263 and without cover = 4215 in 2009 and with cover = 5915 and without cover = 3739 in 2013. Values in percentage of total responded population. ^a^ means the mean difference is significant at the 95% confidence interval

### Effect of dropping PHI cover

Table 4 reports a brief comparison on individual health status and healthcare utilisation before and after dropping PHI cover. The cohort of 193 respondents had PHI cover in 2009 but discontinued it by the time they were interviewed in 2013. The repeated measure t-test results indicate that except for health screening (e.g. Breast cancer: 0.20 vs 0.09, p < 0.05), the mean number of doctor visits (4.6 vs 5.25), hospital admissions (0.11 vs 0.19) and nights’ stay at the hospital (0.34 vs 0.79) did not vary significantly before and after dropping PHI cover. Interestingly, self-assessed health (2.39 vs 2.62, *p* < 0.05) was significantly lower in 2009 compared to 2013, but there was no significant difference in satisfaction with health (7.52 vs 7.21). Lastly, consistent with the findings of Table 2, this cohort of respondents had, on average, a lower number of specialist doctor visits (0.42 vs 0.37), but a higher number of hospital doctor visits (0.27 vs 0.31) after dropping their PHI cover. However, the results were not statistically significant.

**Table 4:** Healthcare utilisation (sub group) before and after dropping private health cover

Healthcare utilisation	Obs	Mean 2009	Mean 2013	p- value
Self-assessed health (1 = excellent; 2 = very good; 3 = good; 4 = fair; 5 = poor)	193	2.39 (0.07)	2.62 (0.08)	0.034
Satisfaction - Your health (0 = totally dissatisfied; 5 = indifferent; 10 = totally satisfied)	192	7.52 (0.12)	7.21 (0.13)	0.086
Household annual expenditure - Fees paid to health practitioners	193	880.9 (95)	679.9 (111)	0.171
Household annual expenditure - Medicines, prescriptions, pharmaceuticals	193	400.6 (33.8)	361.9 (36)	0.436
For most recent doctor visit - any out of pocket expenses for consultation (1 = yes; 2 = no)	160	1.53 (0.04)	1.69 (0.04)	0.002
Number of doctor visits	192	4.6 (0.44)	5.25 (0.61)	0.390
Number of hospital admissions	192	0.11 (0.03)	0.19 (0.04)	0.110
Number of nights in hospital	192	0.34 (0.11)	0.79 (0.21)	0.063
Number of times have you seen your family doctor or GP in the last 12 months	160	5.36 (0.5)	6.30 (0.7)	0.273
Seen during last 12 months - A hospital doctor (i.e., in outpatients or casualty) (0 = no; 1 = yes)	126	0.27 (0.04)	0.31 (0.04)	0.465
Seen during last 12 months - A specialist doctor (excluding in outpatients or casualty of a hospital) (0 = no; 1 = yes)	126	0.42 (0.04)	0.37 (0.04)	0.473
Seen during last 12 months - A mental health professional (0 = no; 1 = yes)	126	0.10 (0.03)	0.19 (0.03)	0.048
During the last 12 months, have you ever been a patient in a hospital overnight? (1 = yes; 2 = no)	192	1.91 (0.02)	1.85 (0.03)	0.081
Had check-up or test in last 12 months - Breast screening (0 = no; 1 = yes)	137	0.20 (0.04)	0.09 (0.03)	0.016
Had check-up or test in last 12 months - Prostate check (0 = no; 1 = yes)	137	0.09 (0.02)	0.07 (0.02)	0.656
Had check-up or test in last 12 months - for bowel cancer (0 = no; 1 = yes)	137	0.06 (0.02)	0.12 (0.03)	0.099
Had check-up or test in last 12 months - Cholesterol test (0 = no; 1 = yes)	137	0.37 (0.04)	0.44 (0.04)	0.000
Had check-up or test in last 12 months - Blood pressure (0 = no; 1 = yes)	137	0.62 (0.04)	0.67 (0.04)	0.082

Obs = number of observations. Standard errors in parenthesis

### Patient background and choice of hospital care

In Table 5, a comparison between the type of hospital care consumed by patients with PHI cover based on their socioeconomic, demographic and lifestyle characteristics is presented. The outcomes of Pearson’s Chi-square tests illustrate that the choice of hospital admission type varies significantly between groups based on age, gender, income levels, and marital status. According to the estimated results of both 2009 and 2013, individuals aged 65 or more (84 among age > 65 vs 67.5 among age < 45 in 2013, *p* < 0.05), from high-income households (annual income > $144,849), or those who are currently married (80.4% among married vs 72.5% among all other in 2013, p < 0.05) were more likely to opt for the private patient option. Moreover, the results of 2013 also indicate that females (25.1 vs 18.8, p < 0.05), patients with BMI lower than 25 (29.3 vs 20.9, p < 0.05), patients without long-term health conditions (25.9 vs 19.1, p < 0.05), smokers (31.5 vs 21.4, *P* < 0.05), patients with higher than average risk-taking attitude (46.2 vs 25.4, p < 0.05), and patients in South Australia were more likely to select public patient care compared to males, patients with higher BMI (> 30), those with long-term health conditions, non-smokers, lower risk-taking attitude and patients in other states, respectively. Experiencing serious personal illness (15.1 vs 11.7, p < 0.05) or financial distress (26.2 vs 11.9, p < 0.05) also influences patients’ choices of hospital care significantly.

**Table 5:** Pearson’s chi square test (public patient vs private patient type admission) for respondents with private health cover

Factors	Valid cases	Private patients (in Public & Private hospital)	Public patient in a public hospital	Pearson Chi-sq	Valid cases	Private patients (in Public & Private hospital)	Public patient in a public hospital	Pearson Chi-sq
	***2009***				***2013***			
**Age**	863			15.75 (0.000)	1196			41.57 (0.000)
Age < 45		69.1	30.9			67.5	32.5	
Age 45–65		74.6	24.5			83.1	16.9	
Age > 65		83.8	16.2			84.0	16.0	
**Education level**	863			1.06 (0.170)	1196			0.70 (0.402)
> High school		76.0	24.0			78.6	21.4	
≤ High school		72.9	27.1			76.5	23.5	
**Household DY**	863			10.01 (0.018)	1196			19.92 (0.000)
Low income		74.0	26.0			79.2	20.8	
Lower middle		70.8	29.2			65.9	34.1	
Higher middle		71.8	28.2			79.0	21.0	
High income		83.4	16.6			81.0	19.0	
**Birthplace**	863			2.42 (0.120)	1187			1.39 (0.237)
Australia		75.0	25.0			76.5	23.5	
Other country		61.5	38.5			80.2	19.8	
**Gender**	863			0.88 (0.347)	1196			6.27 (0.012)
Female		75.8	24.2			74.9	25.1	
Male		73.0	27.0			81.2	18.8	
**Marital status**	863			8.88 (0.003)	1196			10.01 (0.002)
Currently married		78.1	21.9			80.4	19.6	
All other situations		69.0	31.0			72.5	27.5	
**BMI**	863			2.58 (0.460)	1196			10.27 (0.016)
BMI = < 18.5		70.6	29.4			70.7	29.3	
BMI 18.6–24.9		73.5	26.5			74.7	25.3	
BMI 25–29.9		75.4	24.6			81.6	18.4	
BMI= > 30		78.0	22.0			79.1	20.9	
**Remoteness**	863			4.21 (0.040)	1196			0.06 (0.805)
Major city		76.9	23.1			77.5	22.5	
Other places		70.6	29.4			76.8	23.2	
**Long-term health conditions**	862			0.52 (0.471)	1195			7.98 (0.005)
No		73.6	26.4			74.1	25.9	
Yes		75.8	24.2			80.9	19.1	
**Physical activity per week**	764			6.05 (0.048)	1067			0.52 (0.770)
Less than once		73.0	27.0			78.7	21.3	
1–3 times		79.8	20.2			76.5	23.5	
More than 3		71.2	28.8			77.1	22.9	
**Smoking frequency**	860				1052			7.27 (0.026)
Non-smoker						78.6	21.4	
Occasional smoker						63.0	37.0	
Regular smoker						68.2	31.8	
**Self-assessed health**	760			2.04 (0.727)	1065			9.36 (0.530)
Excellent		78.8	21.2			63.9	36.1	
Very good		78.2	21.8			76.6	23.4	
Good		73.3	26.7			78.0	22.0	
Fair		74.5	25.5			80.6	19.4	
Poor		75.9	24.1			80.0	20.0	
**Kessler PDS risk**	768			6.14 (0.105)	1063			3.29 (0.350)
Low		77.6	22.4			79.3	20.7	
Moderate		73.0	27.0			75.7	24.3	
High		73.2	26.8			75.0	25.0	
Very high		61.9	38.1			71.7	28.3	
**Financial risk-taking attitude**					1045			16.04 (0.003)
Never takes risk						74.5	25.4	
Takes average risks						86.9	13.0	
Takes sizeable risks						53.8	46.2	
**Full-time student**	863			0.06 (0.806)	1196			15.31 (0.000)
Yes		76.3	23.7			57.1	42.1	
No		74.5	25.5			78.4	21.6	
**State**	863			5.05 (0.653)	1196			17.24 (0.016)
NSW		75.6	24.4			79.3	20.7	
VIC		78.5	21.5			81.9	18.1	
QLD		72.7	27.3			75.3	24.7	
SA		69.1	30.9			69.1	30.9	
WA		74.2	25.8			79.7	20.3	
**Health shocks**	574			4.39 (0.036)	1054			12.03 (0.001)
Yes		73.2	26.8			84.9	15.1	
No		80.1	19.9			88.3	11.7	
**Financial distress**	574			0.14 (0.71)	1065			35.52 (0.000)
Yes		75.2	24.8			73.8	26.2	
No		77.6	22.4			88.1	11.9	

Note: Data from HILDA survey 2009 and 2013. *P*-values are in the parenthesis. Values in percentage. Here, DY means disposable income. Low income is DY<$63,746), lower middle income is DY = $63,746 to $100,757, higher middle income is $100,758 to $144,848) and high income is DY>$144,849. The variable financial risk-taking attitude was not available in 2009. Tasmania, Northern Territory and Australian Capital Territory had patient count less than 25. Hence, these data are not reported in the table. PDS means psychological distress scale. Identical questions regarding smoking habit and financial risk-taking attitude are not available between 2009 and 2013

Lastly, patients with PHI in 2013 were less likely to choose public hospital care irrespective of income, education, birth origin, gender, marital status and area of residence than patients in 2009. Moreover, the percentage of patients (with PHI) selecting public care reduced considerably across all states from 2009 to 2013 except for South Australia.

### Determinants of the selection of hospital care

The results of the logistic regression model are presented in Table 6. The factors that influence the probability of selecting public hospital care for respondents with PHI cover from 2013 are shown. The reference category for each variable is in parenthesis.

**Table 6:** Key determinants of hospital care-seeking behaviour of patients with private insurance cover

Factors (reference category)	Beta	Wald	S.E.	P-value	Odds ratio
**Self- assesses health (Poor)**					
Excellent	0.039	0.005	0.572	0.942	1.039
Very good	−0.267	0.360	0.460	0.527	0.766
Good	0.031	0.006	0.418	0.937	1.032
Fair	−0.327	0.611	0.445	0.410	0.721
**Household disposable income (High)**					
Low income	0.341	1.324	0.301	0.056	1.407
Lower-middle income	0.591	4.883	0.284	0.032	1.806
Higher-middle income	−0.353	1.718	0.29	0.195	0.703
**BMI (BMI= > 30)**					
BMI < =18.5	0.681	2.666	0.439	0.101	1.976
BMI 18.6–24.9	− 0.039	0.026	0.247	0.858	0.961
BMI 25.29.9	−0.333	1.828	0.253	0.168	0.717
**Age (Age > 65)**					
Age < 45	0.772	6.601	0.302	0.005	2.165
Age 45–65	0.167	0.332	0.282	0.531	1.182
**Type of health cover (Both)**					
Hospital only	0.195	0.506	0.275	0.463	1.215
Extras only	4.053	15.548	2.271	0.001	7.550
**Physical activity (> 3 times a week)**					
< once a week	0.119	0.233	0.255	0.629	1.127
1–3 times a week	−0.119	0.281	0.247	0.614	0.888
**Financial risk-taking attitude (Never)**					
Substantial risks	1.281	3.514	1.616	0.081	3.600
Above average risks	1.56	3.924	5.956	0.04	1.210
Average risks	−0.114	0.148	0.313	0.702	0.892
Not willing	0.026	0.008	0.309	0.922	1.026
**Other compounding variables**					
Born outside Australia (In Australia)	−0.352	1.878	0.267	0.163	0.704
Female (Male)	0.175	0.722	0.21	0.39	1.191
No long-term health condition (Yes)	0.295	1.63	0.245	0.201	1.343
Not a full-time student (Full-time student)	−0.712	3.502	0.417	0.039	0.491
Currently not married (Married)	0.148	0.547	0.22	0.459	1.160
Rural (Urban)	−0.219	0.60	0.326	0.47	0.804
Education more than High school (Otherwise)	−0.447	4.419	0.221	0.033	0.640
Hospital doctor visit (Otherwise)	−0.567	8.415	0.209	0.002	0.567
Specialist doctor visits (Otherwise)	1.183	33.211	0.213	0.001	3.265
Constant	−1.310	3.326	0.769	0.076	0.270
	*Chi-sq*		*P-value*		*R-sq*
Omnibus test model coefficients	252.78		0.000		
Hosmer & Lemeshow	12.99		0.112		
-2 Log likelihood^a^	790.81				
Cox & Snell					0.223
Nagelkerke					0.345

Note: Data from Wave 13. Bootstrap standard errors and p-values. Results are based on 1000 bootstrap samples. Reference category presented in the parenthesis. Dependent variable hospital admission type = 1 if public patient in a public hospital and 0 otherwise

^a^ estimation terminated at iteration number 0.5 because parameter estimates changed by less than 0.001

After adjusting for socioeconomic and demographic characteristics and other key factors, this study found that income level, age, level of education, type of health insurance coverage and type of doctor visits have a significant impact on the selection of hospital care. According to the findings, young patients (age < 45) are 2.2 times more likely to select public care compared to older patients (age > 65). In addition, patients from lower-income (income ≤$100,757) households are 1.4 to 1.8 times more likely to choose public patient care compared to patients from higher-income households (income >$144,849). Conversely, patients with higher education levels (> high school) are 1.56 (1/0.64) times less likely (odds ratio = 0.640, *p* < 0.05) to opt for public patient care in comparison to a patient with lower education levels. Similarly, patients with hospital doctor visits have a lower probability of choosing public patient care (odds ratio = 0.567, p < 0.05). However, patients with higher specialist doctor visits have a 76.55% higher probability of selecting public patient care (the probability has been calculated using the following formula: Pr (Y_p_ > 0) = odds ratio / 1 + odds ratio). Lastly, patients with higher risk-taking attitudes tend to choose public care (1.2 to 1.4 times more) over private care in comparison to patients with lower risk-taking attitudes. All these results are significant at a 95% confidence interval.

Although the following results are statistically insignificant, it is important to note that patients who are women (54%), without long-term health problems (57%), currently not married (53%) and from urban areas (55%) have a higher probability of selecting public care at hospitals compared to patients who are men, with long-term health problems, married and living in rural areas, respectively.

Several diagnostic tests were also conducted, results of which are presented in Table 6 and justify the soundness of the regression model selected. The Omnibus test for model coefficient has *p* < 0.01, which indicates that additional explanatory variables improved the accuracy of the model. The Hosmer and Lemeshow test results suggest that the model is a good fit (*p* > 0.05). The R-square values of Cox and Snell test and Nagelkerke test illustrate that the model explains 22.3 and 34.5% of the variations in the outcome variable, respectively.

The signs and significance of the coefficients were confirmed in the sensitivity analysis (Table 7), further implying the reliability of the model. The results also indicate that patients with PHI in New South Wales (odds ratio 0.366, *p* < 0.05), Victoria (odds ratio 0.270, p < 0.05) and Queensland (odds ratio 0.231, p < 0.05) are significantly less likely to select public hospital care compared to other states.

**Table 7:** Key determinates hospital care-seeking behaviour of patients with private insurance cover (including states)

Factors (reference category)	Beta	Wald	S.E.	P-value	Odds ratio
**Self- assesses health (Poor)**					
Excellent	.199	.134	.545	.715	1.220
Very good	−.218	.229	.455	.633	0.804
Good	.031	.008	.420	.929	1.038
Fair	−.293	.471	.426	.492	0.746
**Household disposable income (High)**					
Low income	.394	1.661	.306	.197	1.483
Lower-middle income	.661	5.773	.275	.016	1.937
Higher-middle income	−.314	1.315	.274	.251	0.730
**BMI (BMI= > 30)**					
BMI < =18.5	.739	2.950	.430	.086	2.094
BMI 18.6–24.9	−.024	.009	.249	.923	0.976
BMI 25.29.9	−.353	1.980	.251	.159	0.702
**Age (Age > 65)**					
Age < 45	.768	6.258	.307	.012	2.155
Age 45–65	.189	.411	.296	.521	1.209
**Type of health cover (Both)**					
Hospital cover only	.310	1.180	.286	.277	1.364
Extra cover only	4.098	8.360	.589	.000	6.204
**Physical activity (> 3 times a week)**					
< once a week	.126	.249	.252	.618	1.134
1–3 times a week	−.159	.476	.230	.490	0.853
**Financial risk-taking attitude (Never)**					
Substantial risks	.867	1.605	.685	.205	2.381
Above average risks	−1.76	4.848	.801	.028	0.171
Average risks	−.196	.422	.302	.516	0.822
Not willing	−.095	.108	.290	.742	0.909
**Other compounding variables**					
Born outside Australia (In Australia)	−.469	3.213	.262	.073	0.626
Female (Male)	.136	.420	.210	.517	1.145
No long-term health condition (Yes)	.315	1.770	.237	.183	1.370
Not a full-time student (Full-time student)	−.615	2.552	.385	.110	0.541
Currently not married (Married)	.216	1.124	.204	.289	1.241
Rural (Urban)	−.128	.201	.285	.654	0.880
Education more than High school (Otherwise)	−.459	4.537	.215	.033	0.632
Hospital doctor visit (Otherwise)	−.556	7.830	.199	.005	0.574
Specialist doctor visits (Otherwise)	1.238	34.843	.210	.000	3.450
**State (Capital Territory)**					
New South Wales	−1.01	4.874	.455	.027	0.366
Victoria	−1.46	9.572	.474	.002	0.231
Queensland	−1.31	7.490	.479	.006	0.270
South Australia	−.392	.592	.510	.441	0.675
Western Australia	−.940	3.538	.500	.060	0.390
Tasmania	−1.46	3.550	.780	.060	0.230
Northern Territory	0.468	.248	.940	.619	1.596
Constant	−.358	.187	.828	0.076	0.699
	*Chi-sq*		*P-value*		*R-sq*
Omnibus test model coefficients	272.462		0.000		
Hosmer & Lemeshow	10.724		0.218		
-2 Log likelihood^a^	771.135				
Cox & Snell					0.24
Nagelkerke					0.37

Note: Data from Wave 13. Bootstrap standard errors and p-values. Results are based on 1000 bootstrap samples. Reference category presented in the parenthesis. Dependent variable hospital admission type = 1 if public patient in a public hospital and 0 otherwise

^a^ estimation terminated at iteration number 0.5 because parameter estimates changed by less than 0.001

## Discussion

This paper provides an estimate of the impact of PHI cover on overall healthcare usage and type of hospital selected among Australian adults using a nationally representative data set. There are significant disparities in secondary preventive care, overnight hospital stay and specialist care utilisations between patients with and without PHI cover. Similar to earlier studies, this study also found that private hospital cover encourages patients to consume private care [6, 23, 34]. This behaviour of PHI patients is understandable as individuals treated as private patients have shorter waiting for treatments, the ability to choose their physicians and enjoy better amenities (e.g. private rooms) [3]. Yet, the results also indicate that around one in four adults in Australia with PHI cover prefers to use public care. Finally, the results of the adjusted binary logistic regression model indicate that lower incomes, younger age, lower levels of education, specialist doctor visits and higher risk-taking attitudes increase the probability of choosing public care among patients with PHI cover. Hence, this study concludes that patients from lower socioeconomic status have a higher probability of choosing public care at the hospitals despite having PHI cover. The critical question is, why?

The Private Health Insurance Act (2007) prohibits insurance providers from discriminating on premium prices based on age, gender, race, religion or health status. However, under this mandatory community rating, premiums are allowed to vary based on the extent of the coverage and treatments included [17, 18]. Young adults are allowed to buy PHI cover while excluding services such as coronary care, joint replacement, cataract surgery and women may decide not to include pregnancy care coverage [41]. People are encouraged to purchase PHI cover due to these exemptions with lower premiums and higher service deductibles along with other “carrot and stick” policies imposed by the government. This explains the findings that people who are younger, female, from low-income households, without long-term health conditions, lower BMI and with higher self-assessed health choose public patient care even though they have PHI cover. Given their comparative good-health or lower ability to pay, they have more probability of buying PHI cover with significant service deductibles compared to people without these characteristics [42]. On the other hand, patients (with or without PHI cover) may choose a private hospital to avoid long waiting times at public hospitals. The expectation of longer waiting times is a significant determinant of a patient taking up PHI cover for private care [10]. Hence, it is justifiable that older patients, patients from high-income households, those with long-term health conditions and lower health status choose private care over public care, regardless of their PHI status. Patients with these characteristics often do not wish or cannot wait a significant time for treatment.

It is also important to note that patients often have little say in the decision to choose the type of hospital care. As Ungar and Ariely [43] indicated, private hospitals in Australia often refer complex patient cases to public hospitals. Patients entering a hospital through emergency departments or for emergency services mostly end up being a public patient [44]. Another important aspect is the lack of information regarding the additional out-of-pocket costs associated with being a private patient at the hospital. The Senate Community Affairs References Committee (2) concluded that patients with PHI cover using care for chronic illness from the private health system bear higher out-of-pocket costs (than those using public care) and are not adequately informed beforehand of the costs. This lack of information may significantly impact the decision of choice of care at the time of needing care. Henceforth, the aforementioned reasons may explain a patient’s decision to use public care.

Similar to previous studies (conducted in other countries), the results indicate that the care choice of patients who suffered life event shocks are different from those without that experience. Findings of these earlier literature [2, 43, 44] partly explain why patients with PHI cover who experienced health shocks or have immediate financial pressure prefer public care over private care.

The estimated results from the merged data of 2009 and 2013 (a cohort of 193 respondents) showed that respondents dropped their PHI cover despite reporting a significant worsening in their self-assessed health status. The mean numbers of health care utilisation (e.g. hospital admissions and hospital nights) were also lower in 2009 (with PHI cover) than in 2013 (dropped PHI cover). These findings nullify the adverse selection hypothesis (people with higher health risk tend to purchase PHI cover) as overall cover consists of a large pool of individuals with lower health risks. This is due to the policies introduced by the federal government (discussed earlier in the section). These findings are similar to the conclusions of earlier comparable studies [5, 6, 17, 45]. A further analysis indicated that in 2013, 74.6% of the respondents with PHI had no long-term health conditions, 49.2% were in the age group of 45 or lower, 87.3% assessed their health as good or higher, 81.8% had a BMI less than 30, 90.3% were non-smokers, and only 23.9% did not do regular exercise (results not reported). Hence, the findings are justifiable.

The evidence also shows that individuals who have PHI cover had a significantly higher rate of health check-ups relative to individuals without it. In addition, a significant disparity was observed in the use of specialist care as patients with PHI (ancillary services coverage) have lower or no out-of-pocket costs of seeing a specialist. These findings uphold the concern raised by previous studies that the PHI system in Australia is inequitable as services are not provided to those who require it, but rather to those who have the ability to pay for it [1, 46].

Finally, the results also indicate that PHI patients who visited hospital doctors are significantly more likely to choose private care and those who visited specialist doctors have a higher probability of selecting public hospital care. It is difficult to explain these findings from the data, and the answer to these findings are well beyond the scope of this study. Hence, future studies could look into the association between PHI status, specialist and hospital doctor visits and the choice of hospital care in Australia.

Several policy suggestions can be offered based on these results. Firstly, it is evident that PHI cover encourages people to use private care. However, a considerable number of PHI patients are not consuming private care when they are eligible may indicate a lack of coherence in the policy and/or consumer information asymmetry, and perceived higher quality and specialisation of public hospitals compared to private hospitals. Besides, proximity to public hospitals may also influence the decision of the patients. Over time, those with specific characteristics (e.g. young age, better health status or low-income) may discontinue PHI, if they assess that they are paying for it without consuming the associated available private services, and if they do then, out-of-pocket costs are higher (than using public care). This trend is evident from the latest AIHW data (Fig. 2). Secondly, respondents with PHI cover showed a notably higher level of health screening than those without. Nonetheless, the rate of screening is less than 30%. PHI providers should encourage their customers to increase the rate of health screening by offering rebates in premiums or expansion in coverage with similar premiums. This preventive behaviour should generate considerable benefits for the health system (private and public) in the long-run. Thirdly, further studies are required to understand why patients from lower socioeconomic status have more probability of using public care despite having PHI cover. It is most likely the out-of-pocket cost associated with using private care, but that has not been proven conclusively. Fourthly, policymakers should examine methods to reduce the inequality in secondary preventive care and specialist care use between PHI patients and those without cover.

This study has some limitations. Firstly, it is difficult to account for any internal factors or policies that govern the PHI provider premiums and coverage policy. Given that the price elasticity of healthcare demand is non-zero, therefore, changes in prices (PHI premiums) have a significant impact on a patient’s decision. Second, the choice between public and private care may be influenced by expectations of the quality of care that will be received and the proximity of a private hospital. This study could not account for these issues. Lastly, since data on the type of disease/illness treated at each hospital for each patient was unavailable, this study could not examine the impact of the type of disease had on the hospital choice decision. On the other hand, the findings indicate that the type of doctor visit significantly influences the hospital choice decision, but it was not clear why. Further studies with primary data are required to understand the relationship between the type of disease, type of doctor visits and choice of hospital care.

## Conclusions

This paper investigated the healthcare use of individuals with or without PHI cover and the determinants of the choice of hospital care (private vs public) of patients with PHI cover. The results indicate that PHI status significantly impacts the use of preventive care, specialist care, and overnight stays at hospitals in Australia. Moreover, patients from lower socioeconomic status (e.g. low income and lower education level) and patients who are relatively young (age < 65), without long-term health conditions, better self-assessed health and had recent experience of serious illness or financial distress have a higher probability of selecting public care at the hospital despite holding PHI cover. Except for specialist care use and the number of hospital night stays, healthcare utilisation did not vary significantly among a cohort of individuals before and after dropping PHI cover. These results are important inputs into policy discussions to enable a more equitable health system, which ensures equal access to care services based on necessity rather than the ability to pay.

## Data Availability

The sources of the data are: This research program prepares and analyses data drawn from the Household, Income and Labour Dynamics in Australia Survey, known as HILDA. Data are available for approved users from the Department of Social Services, Government of Australia. The survey was carried out in accordance with the ethical guidelines approved by University of Melbourne. Details of the data are available at: https://melbourneinstitute.unimelb.edu.au/research-programs/household-income-and-labour-dynamics How to use the HILDA data (user manual): Summerfield, M., Freidin, S., Hahn, M., La, N, Li, N., Macalalad, N., O’Shea, M., Watson, N., Wilkins, R. and Wooden, M. (2016), *‘HILDA User Manual – Release 15’*, Melbourne Institute of Applied Economic and Social Research, University of Melbourne.

## Abbreviations

AIHW: Australian Institute of Health and Welfare
BMI: Body Mass Index
GDP: Gross Domestic Product
GP: General Practitioner
HILDA: Household, Income and Labour Dynamics in Australia
OECD: Organisation for Economic Co-Operation and Development
PHI: Private Health Insurance
Pr: Probability
